# Renal Cortical Necrosis Secondary to Idiopathic Acute Pancreatitis in a Young Adult: A Case Report

**DOI:** 10.7759/cureus.94212

**Published:** 2025-10-09

**Authors:** Ram Prabahar M, Raniya Palliyedath, Jayanivash Jayam, Sathiyan Sivanandam, Uma Sirisha Pusapati

**Affiliations:** 1 Department of Nephrology, SIMS Hospitals, Chennai, IND; 2 Department of Internal Medicine, SIMS Hospitals, Chennai, IND

**Keywords:** acute kidney injury, acute pancreatitis, end-stage kidney disease, hemodialysis, renal cortical necrosis

## Abstract

Renal cortical necrosis (RCN) is a rare and severe form of acute kidney injury (AKI), often resulting in irreversible kidney failure. We present the case of a 25-year-old male who developed severe acute pancreatitis (AP), leading to dialysis-requiring AKI. Despite remission of the initial AP episode, he remained anuric and dialysis-dependent for four weeks. A renal biopsy revealed diffuse cortical necrosis. He was continued on maintenance hemodialysis via a right internal jugular vein (IJV) tunneled venous catheter. Eight weeks after the initial episode, he presented with acute abdominal pain. Follow-up imaging studies showed gallbladder sludge and signs of walled-off pancreatic necrosis; endoscopic and surgical procedures (endoscopic ultrasound (EUS)-guided cystogastrostomy and laparoscopic cholecystectomy) were performed. However, he experienced repeated episodes of AP over the next 18 months. Genetic testing was negative, and the etiology was deemed idiopathic. Each episode was managed conservatively. He has been wait-listed for deceased donor renal transplantation and continues maintenance hemodialysis. Our patient developed dialysis-requiring AKI during the first episode of AP, secondary to RCN, and progressed to end-stage kidney disease (ESKD). He is currently awaiting renal transplantation. This case highlights the range of multi-organ dysfunction that can be associated with severe AP and its progression to chronic kidney disease.

## Introduction

Acute pancreatitis (AP) is an inflammatory condition of the pancreas, typically presenting with acute abdominal pain. Disease severity is variable: mild AP is characterized by the absence of organ failure and local complications, moderate AP by transient organ failure resolving in less than 48 hours, and severe AP by persistent multi-organ failure [1]. Organ failure is commonly assessed using the modified Marshall scoring system, which evaluates the cardiovascular, respiratory, and renal systems. Acute kidney injury (AKI) complicates 9% to 12% of severe AP cases and is strongly associated with poor prognosis, particularly when kidney replacement therapy (KRT) is required. The need for KRT in AKI secondary to AP is variable, ranging from 26% to 63% in several series [2]. The pathophysiology is multifactorial, ranging from initial volume depletion to complex vascular and humoral factors. RCN is a rare subset of AKI, with a prevalence of 1.9% to 2% in developed countries compared with 6% to 7% in developing regions [3]. Although RCN is usually associated with obstetric catastrophes or sepsis, its occurrence in idiopathic AP is rarely documented, underscoring the clinical significance of this case.

## Case presentation

**Figure 1:**
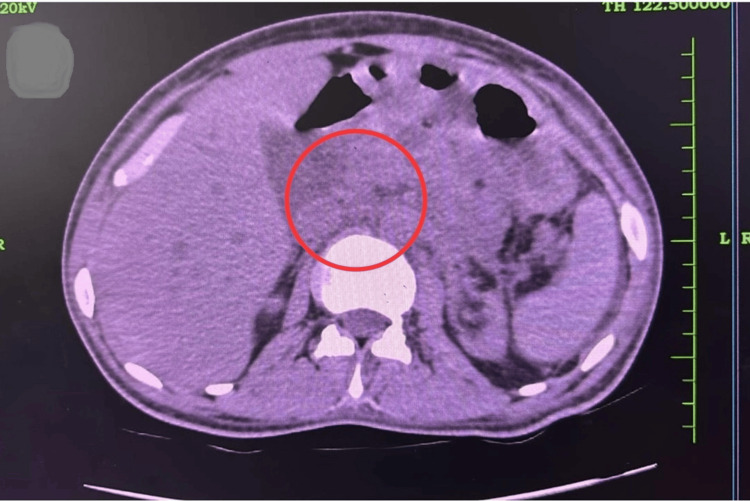
Computed tomography (CT) scan of the abdomen showing an edematous uncinate process and proximal pancreas with fat stranding.

A 25-year-old man with no prior comorbidities presented with severe epigastric pain radiating to the back, persistent vomiting, and oliguria. He denied smoking, alcohol, or substance use. He was hypovolemic with low-normal blood pressure on admission. Laboratory investigations revealed azotemia, anemia, markedly elevated amylase and lipase, and elevated C-reactive protein (CRP) (Table 1). Imaging showed an edematous pancreas with peri-pancreatic inflammation and normal-sized kidneys bilaterally, with minimal perinephric stranding (Figure 1). He developed severe third-space fluid collections, including ascites, bilateral pleural effusion, and pericardial effusion. He was admitted to the intensive care unit and managed conservatively with bowel rest, intravenous fluids, and total parenteral nutrition. Hemodialysis was initiated for AKI.

**Table 1:** Laboratory parameters at the time of presentation. The bold values indicate the results that are outside the normal range in the table. PCV, packed cell volume; RBC, red blood cell; MCV, mean corpuscular volume; WBC, white blood cell; CRP, C-reactive protein; LDH, lactate dehydrogenase; SGOT, serum glutamic oxaloacetic transaminase; AST, aspartate aminotransferase; SGPT, serum glutamic pyruvic transaminase; ALT, alanine aminotransferase; Na⁺, sodium; K⁺, potassium; Cl⁻, chloride; RBCs, red blood cells; C3, complement component 3; C4, complement component 4; ANA, antinuclear antibody; ANCA, antineutrophil cytoplasmic antibody; dsDNA, double-stranded deoxyribonucleic acid; SPEP, serum protein electrophoresis; UPEP, urine protein electrophoresis; ADAMTS13, a disintegrin and metalloproteinase with thrombospondin motifs 13

Parameter	Patient value	Reference range
Hemoglobin	8.3 g/dL	13-17 g/dL
PCV (Hematocrit)	20.9%	40%-50%
RBC count	2.07 × 10⁶/µL	4.5-5.9 × 10⁶/µL
MCV	101 fL	80-100 fL
Total WBC count	17,090 /µL	4,000-11,000 /µL
Neutrophils	89%	40%-75%
Lymphocytes	16%	20%-45%
Eosinophils	3%	<6%
Absolute neutrophil count	15,120/µL	1,500-8,000/µL
Platelet count	428,000/µL	150,000-450,000/µL
Peripheral smear	No evidence of hemolysis; neutrophilia noted	-
Serum amylase	1,200 U/L	30-110 U/L
Serum lipase	2,500 U/L	0-160 U/L
C-reactive protein (CRP)	160 mg/L	<10 mg/L
LDH	220 U/L	140-280 U/L
Total bilirubin	4.03 mg/dL	0.2-1.2 mg/dL
SGOT (AST)	348 U/L	<40 U/L
SGPT (ALT)	186 U/L	<40 U/L
Alkaline phosphatase	135 U/L	45-120 U/L
Serum creatinine	14.49 mg/dL	0.7-1.3 mg/dL
Blood urea	129 mg/dL	7-20 mg/dL
Sodium (Na⁺)	139 mmol/L	135-145 mmol/L
Potassium (K⁺)	4.3 mmol/L	3.5-5.0 mmol/L
Chloride (Cl⁻)	104 mmol/L	98-106 mmol/L
Calcium	8.7 mg/dL	8.5-10.5 mg/dL
Urine routine/microscopy	Clear, specific gravity 1.020, no protein, no RBCs, no casts	-
Complement levels	C3: 125 mg/dL; C4: 32 mg/dL	C3: 90-180; C4: 10-40 mg/dL
Serologies/immune markers	ANA, ANCA, dsDNA - Negative	-
ANA profile (Immunoblot)	Negative	-
Paraprotein workup	SPEP/UPEP - No M band	-
ADAMTS13 activity	65%	>50%
Triglycerides	175 mg/dL	150-199 mg/dL
Total cholesterol	79 mg/dL	<200 mg/dL

**Figure 2:**
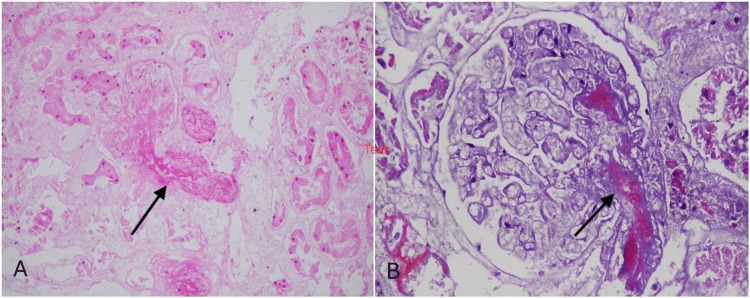
(A) Diffuse coagulative necrosis of renal cortical tissue with ghost-like glomeruli and tubules (hematoxylin and eosin (H&E) stain, 100×), and (B) glomerulus with fibrin thrombus occluding the efferent arteriole alongside necrotic surrounding tubules (H&E stain, 400×).

Although the initial AP episode resolved, the patient remained anuric. He remained dialysis-dependent at three weeks, after which a renal biopsy was performed. The biopsy revealed diffuse cortical necrosis characterized by complete loss of viable cortical tissue, necrotic glomeruli, and fibrin thrombi occluding efferent arterioles and interlobular arteries (Figure 2). The first AP episode had a severe impact on the cardiovascular system, with the patient developing dilated cardiomyopathy and global hypokinesia. Due to a low ejection fraction (30%), he was continued on maintenance hemodialysis via a right internal jugular vein (IJV) tunneled venous catheter.

**Figure 3:**
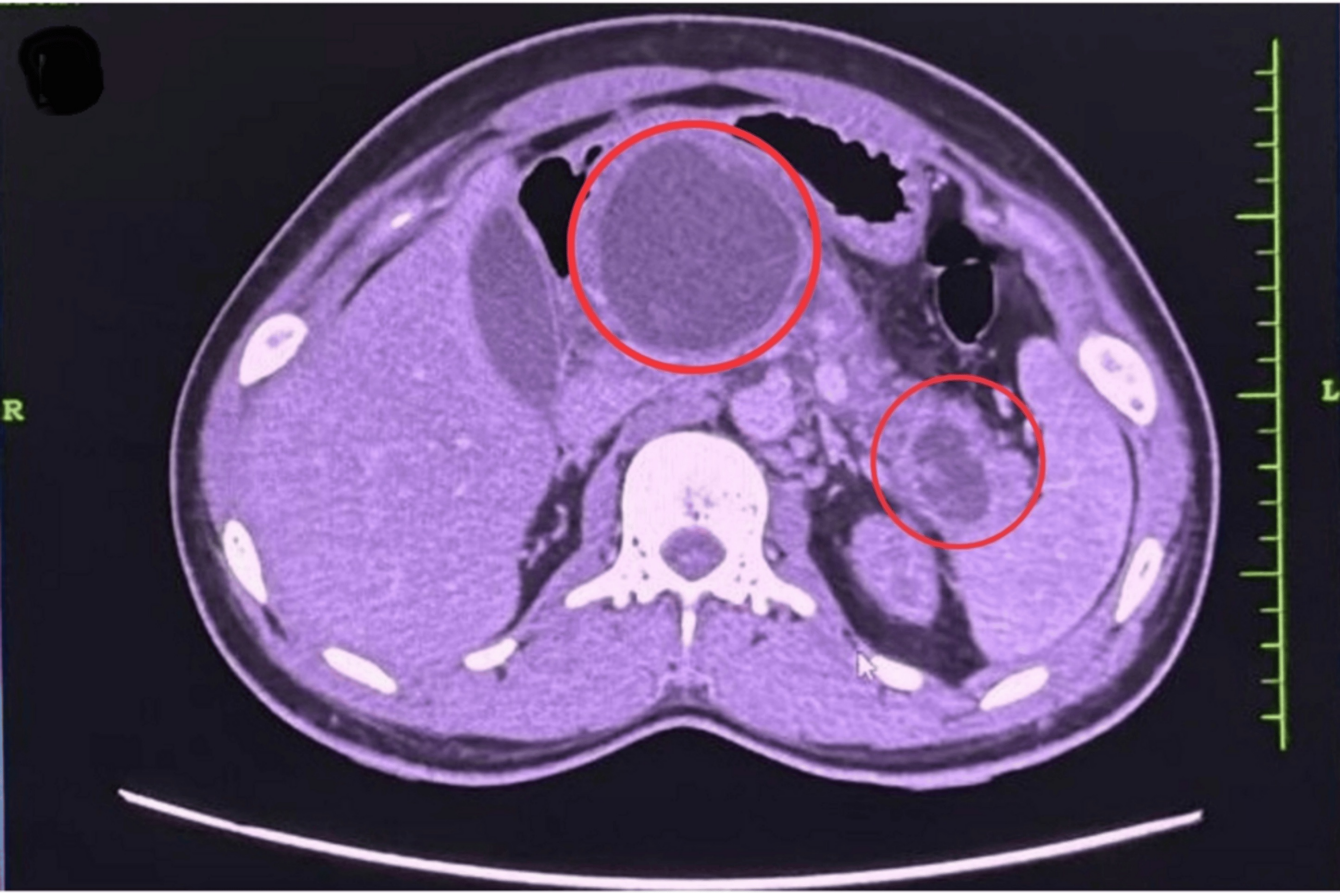
CT abdomen showing a well-defined, encapsulated fluid collection with heterogeneous internal contents, consistent with walled-off pancreatic necrosis (WOPN).

He was hospitalized with a second episode of AP eight weeks after the first. Follow-up imaging revealed gallbladder sludge and walled-off pancreatic necrosis (Figure 3). He underwent EUS-guided cystogastrostomy with placement of a 15-mm lumen-apposing metallic stent, followed by laparoscopic cholecystectomy. Both procedures were completed without complications. The biopsy specimen did not show gallstones or cholecystitis.

Over the subsequent 18 months, he experienced recurrent episodes of AP. During this period, he developed endocrine pancreatic insufficiency and type 3c diabetes mellitus (T3cDM). There were no clinical or laboratory features suggestive of thrombotic microangiopathy. Evaluation for recurrent AP, including serum triglycerides, calcium, IgG4, abdominal CT, and EUS-guided analysis, was unremarkable. Comprehensive genetic testing, including PRSS1 (serine protease 1, cationic trypsinogen), SPINK1 (serine peptidase inhibitor Kazal type 1), CFTR (cystic fibrosis transmembrane conductance regulator), and CTRC (chymotrypsin C), was negative, supporting the classification of AP as idiopathic. He remains dialysis-dependent and is currently awaiting deceased donor renal transplantation.

## Discussion

Severe AP often leads to multi-organ failure and poor outcomes, with AKI being a well-recognized complication. AKI complicates 9% to 12% of AP cases and is associated with significantly increased mortality, with rates as high as 30% reported in one series [1,4]. The etiology of AKI is multifactorial, including hypovolemia, systemic inflammation, toxins from the necrotic pancreas, and abdominal compartment syndrome [5]. Renal recovery usually parallels the resolution of pancreatic inflammation, though 15% to 20% of patients progress to chronic kidney disease. Treatment is essentially supportive, with KRT required in severe cases. Our patient developed AKI during the first AP episode and progressed to end-stage kidney disease (ESKD).

RCN is a severe form of AKI, often resulting in irreversible renal failure. It is defined by total ischemic necrosis of all nephron elements. Diffuse forms lead to irreversible failure, while patchy forms may allow partial recovery. It typically results from ischemia due to reduced perfusion, intravascular coagulation, microvascular injury, or severe vasospasm. Etiologies are classified as obstetric or non-obstetric, with septic abortion and hemolytic uremic syndrome being the most common causes in each group. In developing countries, such as India, the incidence of RCN has declined over recent decades, largely due to reduced rates of unsafe abortions [6,7].

The pathogenic mechanism remains incompletely understood, though small-vessel vasospasm and toxin-induced endothelial injury are considered initiating events. While obstetric complications and sepsis are the predominant causes of RCN, its occurrence as a complication of AP remains exceptionally rare, with fewer than a dozen cases described. Most cases resulted in irreversible renal injury progressing to ESKD [8-10]. In addition to the usual pathogenic factors, vasoactive and cytotoxic substances released during AP are thought to contribute. In line with the systemic impact of severe AP, our patient also developed walled-off pancreatic necrosis requiring drainage and later developed chronic dilated cardiomyopathy with reduced ejection fraction.

## Conclusions

Severe AP can cause significant multi-organ damage. This case highlights the rare occurrence of RCN secondary to AP in a young patient, leading to irreversible, dialysis-dependent ESKD. It underscores the importance of early recognition and vigilant management of renal complications in AP, as well as the need for long-term follow-up given the potential for recurrent episodes, chronic morbidity, and eventual renal transplantation.
